# Acceleration-induced pressure gradients and cavitation in soft biomaterials

**DOI:** 10.1038/s41598-018-34085-4

**Published:** 2018-10-26

**Authors:** Wonmo Kang, Marc Raphael

**Affiliations:** 1 Leidos, Arlington, VA 22203 USA; 2 Naval Research Laboratory, Washington, DC 20375 USA

## Abstract

The transient, dynamic response of soft materials to mechanical impact has become increasingly relevant due to the emergence of numerous biomedical applications, e.g., accurate assessment of blunt injuries to the human body. Despite these important implications, acceleration-induced pressure gradients in soft materials during impact and the corresponding material response, from small deformations to sudden bubble bursts, are not fully understood. Both through experiments and theoretical analyses, we empirically show, using collagen and agarose model systems, that the local pressure in a soft sample is proportional to the square of the sample depth in the impact direction. The critical acceleration that corresponds to bubble bursts increases with increasing gel stiffness. Bubble bursts are also highly sensitive to the initial bubble size, e.g., bubble bursts can occur only when the initial bubble diameter is smaller than a critical size (≈10 μm). Our study gives fundamental insight into the physics of injury mechanisms, from blunt trauma to cavitation-induced brain injury.

## Introduction

The transient dynamic response of biological materials during rapid mechanical loading^1–3^ is becoming increasingly important due to emerging medical implications, i.e., injury mechanisms under blast, ballistic, or impact exposures^4–6^. When exposed to these threats, a biological system, e.g., human brain or skin, is rapidly accelerated, which results in the acceleration-induced pressure gradient. Depending on the amplitude and duration of such mechanical inputs, the biological system may be subjected to everything from small deformations to considerably larger damage associated with sudden bubble bursts. For the accurate prediction and assessment of potential injuries, a fundamental understanding of how soft materials respond under rapid loading conditions is critical.

Despite this persistent need, the underlying mechanisms that govern biomaterial deformation are not fully understood, mainly due to the complex coupling of *fluid-* and *solid-like* characteristics. In addition, accurate characterization of soft biomaterial properties, including biological hydrogels or tissues, remains challenging due to their soft, labile nature^7–10^, nonlinear material response^11–15^, and strain-rate-dependent material properties^16,17^.

As an example, while it is well known that material properties of soft materials greatly depend on their concentration as well as loading conditions, experimental measurements on the key properties such as surface tension and elasticity, in particular, at fast loading conditions are still very limited. Established experimental techniques^18^ to quantify surface tension are mostly for *liquid-phase* solutions, and as a result, the induced surface tension for *gel-phase* samples (i.e., fully polymerized hydrogels) is not well characterized. It is worth noting that there are several emerging technologies specifically designed for measuring the surface tension of soft materials^19–21^. For elastic modulus measurements, many characterization techniques^8,9,22^, including high loading conditions^17^, are readily available for soft material samples, but the quantitative values for soft biological tissue samples (e.g. elastic modulus) can significantly vary depending on the characterization methods^23^.

One notable advance is in the characterization of cavitation properties for soft materials under an impulsive force^15,24^ where the critical acceleration that corresponds to the onset of the bubble formation and bursts is quantitatively measured. While the acceleration-induced pressure is the primary driving force for this rapid material deformation associated with bubble dynamics in pure water and gelatin samples, the spatio-temporal dynamics in soft material samples during mechanical impact remains largely unknown.

Here we experimentally and theoretically consider transient bubble dynamics in biologically-relevant soft materials, collagen and agarose, that are commonly used as an extracellular matrix (ECM) for 3-dimensional (3D) cell culture. For quantitative characterization of soft materials, we utilize a recently developed experimental setup that is based on a drop tower system^10,15^. First, we directly visualize the acceleration-induced pressure gradient during impact utilizing 1% collagen samples with optically visible, macro air bubbles. By analyzing high speed camera images, the maximum bubble size is correlated to the mechanical input and sample height while monitoring key bubble behaviors, including burst and collapse. We further quantify the critical mechanical inputs that trigger cavitation nucleation and bursting in agarose samples as a function of the sample stiffness. Finally, we develop a theoretical model for predicting transient bubble dynamics.

## Results

**Figure 1:**
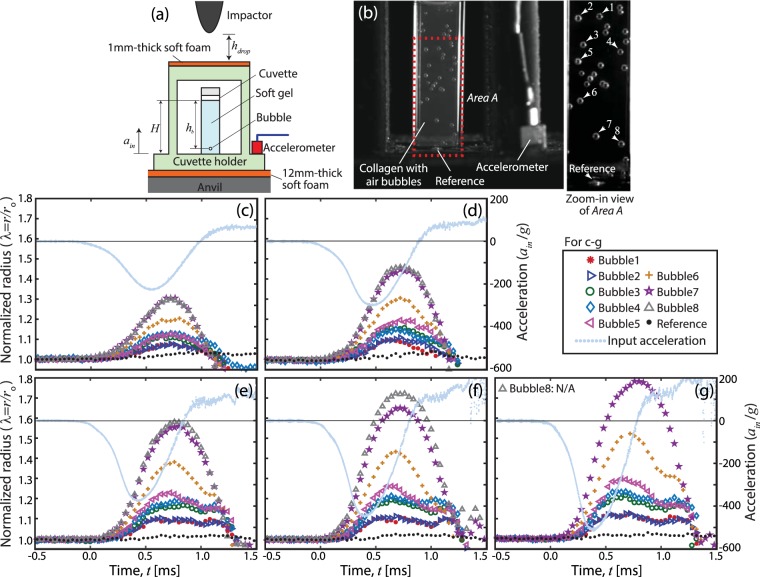
Deformation of air bubbles in a soft gel due to acceleration-induced pressure gradient during mechanical impact. (**a**) shows schematic of a sample holder under a drop-tower system for the characterization of soft material samples in a cuvette where *h*_*drop*_, *H*, and *h*_*b*_ are the drop height, sample height, and bubble location with respect to the gel’s top surface, respectively. Rigid-body acceleration (*a*_*in*_) of the cuvette holder was measured by the accelerometer during impact. (**b**) is a high speed camera image of a 1% collagen sample in a transparent cuvette before impact. The zoom-in view of *Area A* shows eight target air bubbles (1–8) and a rigid plastic inclusion at the bottom of the cuvette (*Reference*). Acceleration signals normalized by gravity (***g***) in c-g (vertical axis on the right) corresponded to 4, 6, 8, 10, and 12 cm drops, respectively. By performing image analysis of high speed image frames in time (*t*), the radii (*r*) of the individual target bubbles indicated by the arrows (1–8 in **b**) were monitored. (**c**–**g**) show the normalized radius (*λ* = *r*/*r*_*o*_) of the bubbles and reference (vertical axis on the left) due to the impact at *t* = 0 where *r*_0_ is the initial radius. (**a**) is modified from^15^.

We experimentally considered transient bubble dynamics in soft material samples during mechanical impact. For this study, a series of drop tower experiments were performed in which the amplitude of each impact was controlled by changing vertical height (*h*_*drop*_) of a movable mass (impactor) with respect to the customized sample holder (Fig. 1a). The holder was designed to accommodate a transparent cuvette containing the soft material sample. Once released, the impactor was accelerated toward the holder by gravity (*g*) until collision. During impact, high speed cameras and an accelerometer were utilized for concurrent visual observation and impulse force quantification. Detailed sample preparation and experimental procedures are discussed in the Methods section.

It is important to note that a soft foam sheet is placed between (1) the sample holder and rigid anvil as well as (2) the impactor and sample holder, respectively (see Fig. 1a). Because these foams are readily deformable upon mechanical impact, the sample holder is free to move vertically against the foam stiffness. In addition, these foams prevent a direct collision between the rigid parts including the holder, anvil, and impactor, which would result in generating shock waves and propagating waves to the sample via surrounding solid structures, i.e., the sample holder and cuvette. By utilizing the soft foam sheets, we achieve the smooth acceleration profiles, which are critical to study acceleration-induced cavitation, as shown in Fig. 1c–g without any pulse-like signals. In-depth dynamics analysis of the experimental setup can be found elsewhere^10^.

### Acceleration-induced pressure gradient and transient bubble dynamics

Here we focus on the characterization of acceleration-induced pressure gradients in soft materials during impact. For direct visualization of the pressure gradient we utilized collagen, the most abundant extracellular protein *in vivo* and one of the most commonly used proteins for 3D cell culture^25–27^, prepared in a transparent cuvette with randomly distributed, macro air bubbles (Fig. 1b). To quantify the correlation between bubble size and input acceleration, bubble radii (*r*), indicated by the arrows in Fig. 1b, were measured by performing image analysis of each high speed image frame (see the Methods section). Next, the bubble size was correlated with the acceleration measurements as shown in Fig. 1c–g.

Figure 1c–g show the normalized bubble radius (*λ* = *r*/*r*_0_, vertical axis on the left) utilizing 5 different drop heights, i.e., *h*_*drop*_ = 4, 6, 8, 10, and 12 cm, respectively. The corresponding acceleration signals induced by these mechanical impacts at *t* = 0 are also shown (vertical axis on the right) where the amplitudes of the acceleration, |*a*_*amp*_|, were 228.52, 300.78, 376.95, 448.24, and 516.60 *g*, respectively, and *t* is time. To measure the initial radius (*r*_0_), the mean average of each target bubble was obtained over 49 high speed image frames from *t* = −2 ms to  = 0 ms (see Table S1 for the initial radius (*r*_0_) and vertical location (*h*_*b*_) of each bubble with respect to the top surface of the collagen sample (Fig. 1a) where the average bubble radius of all 8 bubbles was 0.52 mm). As expected, before the impact (*t* < 0), *r*/*r*_0_ = 1 for all bubbles. Supplementary Movies 1–5 show the acceleration measurement and corresponding high speed camera images for Fig. 1c–g, respectively.

The results in Fig. 1 indicate that an increase in bubble size depends on not only the drop height (*h*_*drop*_), but the vertical location (*h*_*b*_) of each bubble. For example, with regards to drop height dependence, the maximum normalized radius (*λ*_*max*_ = *r*_*max*_/*r*_0_) of *Bubble 7* increased by 40% when drop height increased from 4 cm to 12 cm (Fig. 1c,g, respectively). With regards to vertical location, bubbles of the same initial radii, for example *Bubble 1* at the top of the field of view and *Bubble 8* at the bottom, exhibited radically different deformations with *λ*_*max*_ = 1.08 for *Bubble 1* and *λ*_*max*_ = 1.31 for *Bubble 8* (4 cm drop height). This effect only became more pronounced as the drop height increased.

**Figure 2:**
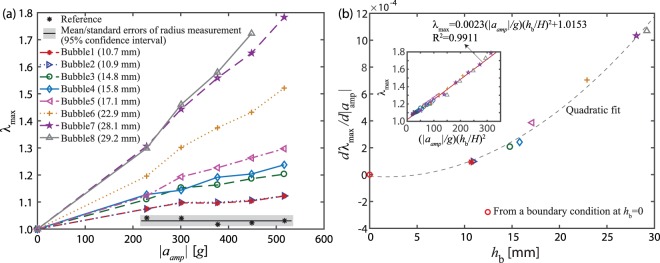
The correlation between the amplitude of input acceleration (|*a*_*amp*_|) and the corresponding normalized maximum radius (*λ*_*max*_ = *r*_*max*_/*r*_0_) of bubbles at different depth of bubbles (*h*_*b*_) where *r*_*max*_ and *r*_0_ are the maximum and initial radii, respectively. The *λ*_*max*_ and |*a*_*amp*_| relation in (**a**) indicates that *λ*_*max*_ is linearly proportional to |*a*_*amp*_| while each slope, *dλ*_*max*_/*d*|*a*_*amp*_|, depends on *h*_*b*_. In (**b**), the slopes for each *λ*_*max*_ and |*a*_*amp*_| curve are shown as a function of *h*_*b*_. Note that pressure at *h*_*b*_ = 0 is expected to be zero to satisfy a free surface boundary condition. The inset in b confirms the ${\lambda }_{max}-1\propto |{a}_{amp}|{h}_{b}^{2}$ relation using the least square fitting method.

To quantitatively characterize the correlation between *λ*_*max*_, |*a*_*amp*_|, and *h*_*b*_, we plotted *λ*_*max*_ for each bubble as a function of |*a*_*amp*_| in Fig. 2a, which shows that the slopes of the *λ*_*max*_ and |*a*_*amp*_| curves strongly depend on *h*_*b*_. Furthermore, we found that the slope is proportional to ${h}_{b}^{2}$ utilizing the *linear least-squares* fitting in Fig. 2b. Based on these experimental observations, we concluded that *λ*_*max*_ is linearly proportional to (|*a*_*amp*_|/*g*)(*h*_*b*_/*H*)^2^ as summarized in the inset of Fig. 2b, i.e., *λ*_*max*_ = 0.0023(|*a*_*amp*_/*g*|)(*h*_*b*_/*H*)^2^ + 1.0153 with *R*^2^ = 0.9911 where *H*, the total height of the collagen sample (Fig. 1a), was 36 mm. It is worth noting that dynamic size characterization of individual bubbles became increasingly difficult with increasingly higher drops because bubble became large enough to directly interact with either other bubbles or cuvette walls. As an example, the radius of *Bubble 8* is not available Fig. 1g because it merged with a neighboring bubble (see Supplementary Movie 5).

Another important observation from Fig. 2a is that bubble bursts, a sharp increase in bubble radius (*λ*_*max*_ ≫ 1) with a small increase in a mechanical input, were not observed even for a considerably large input acceleration (=516.60 *g*). This is interesting because it was reported that input accelerations of ~500 *g* triggers bubble bursts even in much stiffer gelatin samples *without macro bubbles*^15^, e.g., the nominal shear moduli for 7.5% gelatin^28^ and 1% collagen^9^ are approximately 20  kPa and 10 Pa, respectively, at relatively low strain rates. In general, the higher stiffness results in increasing resistance against bubble expansion and, as a result, requires higher accelerations to trigger bubble bursts. Because our observations are contradictory to this statement, we hypothesized that, in addition to material stiffness, bubble burst in soft material samples is also dependent upon initial bubble size, as is further discussed below.

For validation of our experimental approach, we also measured the size of the *reference* (*r*_*ref*_), a non-circular solid, plastic inclusion at the bottom of the cuvette. Unlike bubbles, the reference was rigid and, as a result, *r*_*ref*_/*r*_0_ ≈ 1 regardless of drop height. The small fluctuation in *r*_*ref*_/*r*_0_ observed for *t* > 0 was likely due to illumination variations as the cuvette holder moved vertically with respect to the lights during impact. We estimated standard error in size of the reference when |*a*_*amp*_| > 0 (Fig. 2a) and found that the mean and 95% confidence interval were *r*_*ref*_/*r*_0_ = 1.03 ± 0.02 (indicated by the gray area). This confirmed a vibration induced measurement error which was significantly smaller than *λ*_*max*_ values for the bubbles and, therefore, we concluded that our observations/analysis on the bubble size are not due to measurement artifacts.

### Bubble burst in soft materials

For the burst of bubbles in soft materials, we utilized agarose samples, another common material for 2D/3D cell culture^29,30^, for two main reasons: (1) agarose is considerably stiffer than collagen and hence we can explore bubble dynamics for a wide range of material stiffnesses and (2) unlike collagen, agarose samples can be readily prepared without pre-existing macro bubbles. Mechanical stiffness of biological samples typically covers a broad range from 0.1~10  kPa for brain tissues^31–33^ to 100  kPa for human aorta tissue^34^. Agarose with its concentration dependent stiffness can be easily tuned to mimic various target tissues, offering an attractive advantage as a tissue simulant. Second, optically visible bubbles (>1 μm) generally do not exist in biological systems such as tissues and organs. Therefore, characterizing gels without macro bubbles during impact is important for understanding potential bubble formation, also known as cavitation nucleation, in these systems and the possible damage mechanisms associated with cavitation nucleation, bubble burst, and collapse.

For this study, we first characterized the critical acceleration (*a*_*cr*_) that corresponds to cavitation nucleation followed by bubble burst in the agarose samples. To do so we prepared agarose samples without optically visible bubbles and detected bubbles that nucleated directly as a result of impact. Eighteen agarose samples at each concentration (0.3%, 0.58%, 0.9%, and 1.5% agarose) were prepared in individual cuvettes following the protocol discussed in the Methods section. The onset of cavitation was detected by visual observation of the high speed camera video of the impacted sample. Note that the smallest bubble radius that was optically detectable was on the order of ~100 μm due to the limited spatial resolution of the high speed camera images.

**Figure 3:**
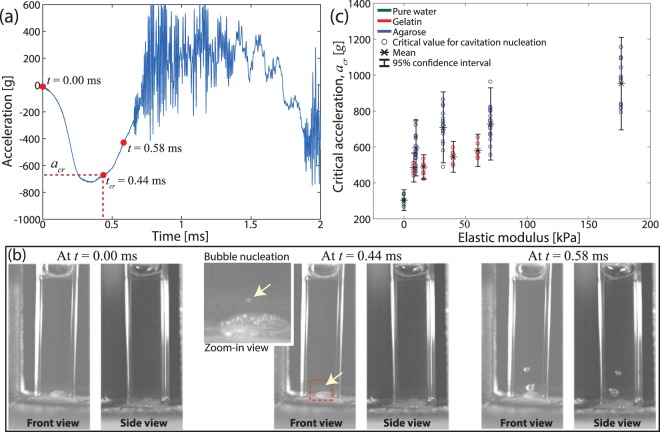
The critical acceleration (*a*_*cr*_) for the onset of cavitation nucleation in soft samples. (**a**) Acceleration signal for a 0.3% agarose sample during impact and **(b**) the corresponding high speed camera images at three different time points: (left to right) initial configuration, cavitation nucleation, and the maximum radii of bubbles. (**c**) shows *a*_*cr*_ as a function of the estimated elastic modulus of soft material samples. The critical acceleration of agarose is directly compared with pure water (green) and gelatin (red) samples^15^.

Figure 3a,b show acceleration measurement and the corresponding material response for a 0.3% agarose sample, respectively, during impact (see Supplementary Movie 5). In Fig. 3b, no bubble was optically detected at *t* = 0 ms and then a cavitation bubble (see the zoom-in view) was first observed at *t*_*cr*_ = 0.44 ms. Finally *t*_*cr*_ in Fig. 3a was directly correlated to the acceleration signal to determine the corresponding acceleration (*a*_*cr*_). The same experimental procedure above was repeated for all 72 agarose samples to characterize cavitation properties (see Tables S2–5 for the detailed data). Supplementary Movies 6–9 show representative experimental results for 0.3%, 0.58%, 0.9%, and 1.5% agarose, respectively. To minimize the possible effect of accumulated damage to samples on the measured critical acceleration values, we replaced a sample by a new one whenever cavitation nucleation was detected.

Figure 3c shows the critical acceleration as a function of elastic modulus of agarose samples where ${E}_{a}=85{c}_{a}^{1.8}\,{\rm{kPa}}$^16^ was used to convert agarose concentration (*c*_*a*_) to elastic modulus (*E*_*a*_). Similarly, cavitation properties of gelatin samples^15^ were also converted using *E*_*g*_ = 8*c*_*g*_  kPa^28^ for direct comparison. In using these relationships, we note the that static elastic modulus of soft gels as a function of gel concentration is well established in the literature^16,28^. Also, it is known that elastic properties within small material deformation of gels are relatively insensitive to strain rates at low strain rates (~10/min)^35,36^ as well as high strain rates (~1000/s)^37,38^. In addition, previous studies on laser- as well as acoustically-induced cavitation bubbles in gels showed that use of strain-rate independent nominal elastic modulus captures bubble dynamics reasonably well^14,39^.

Both agarose and gelatin samples quantitatively show a similar trend in increasing the critical acceleration with increasing elastic modulus, i.e., a sharp increase from pure water to ~10  kPa followed by a more modest slope for higher elastic modulus. This trend can be explained by (1) concentration-dependent material properties of soft gels and (2) interplay between pre-existing material defects, e.g., micro/nano-bubbles, and gel molecules. As briefly discussed earlier, gel stiffness including collagen, agarose, and gelatin increases with increasing concentration^16,22,28^. For stiffer gels, more energy is needed for local deformation of a gel associated with bubble expansion and, as a result, larger mechanical inputs, i.e., higher accelerations, is expected to trigger cavitation nucleation and bubble bursts.

Regarding the bubble-gel interplay in gels without macro bubbles, small traces of nanoscale air bubbles (~hundreds nanometers in size) in ultrapure water have been detected utilizing dynamic light scattering^40^. Also it is known that dissolved gases in water tend to accumulate at the interfaces between solids and water to form nanoscale bubbles^41,42^. Because all samples, including agarose, gelatin, and pure water, in Fig. 3c were prepared using ultrapure water and had either a cuvette-gel or -water interface, submicron-scale bubbles, although optically invisible, likely existed in the samples.

For pure water, the shape of a bubble is mainly determined by surface energy minimization, i.e., a sphere, because water is a rigidity-free liquid medium. Unlike pure water, a bubble (or material defect) in gels would directly interact with the neighboring gel structures. Due to gel stiffness, a bubble cannot change its shape without deforming the gel structures and, as a result, the bubble shape at equilibrium is determined by surface energy of a bubble as well as stored-strain energy in gel. As a result, a bubble can be non-spherical, which increases its area-to-volume ratio. It was theoretically shown that a bubble with a larger area-to-volume ratio requires higher mechanical inputs for cavitation nucleation^15^, explaining the sharp increase in *a*_*cr*_ from pure water to the lower concentrations of gels in Fig. 3c.

Compared to collagen with macro bubbles, we noticed distinctively different dynamic responses in agarose, gelatin, and pure water without macro bubbles. While the maximum normalized radius of each bubble is linearly proportional to the amplitude of acceleration for collagen (*λ*_*max*_ ∝ |*a*_*amp*_|), the bubble dynamics in the agarose, gelatin, and pure water samples was highly non-linear and stochastic. As an example, no bubbles were optically observed when *t* < *t*_*cr*_ and then millimeter scale bubbles suddenly appeared for *t* > *t*_*cr*_. Also, the maximum bubble radius in the agarose sample in Fig. 3b was *r*_*max*_ = 1.13 mm at *t* = 0.58 ms while the smallest bubble radius that was optically detectable was *r* = 180 μm at *t*_*cr*_ = 0.44 ms (Fig. 3b). This result indicates that *λ*_*max*_ values for the micro/nano-scale bubbles are expected to be much larger than macro bubbles, e.g., *λ*_*max*_ for agarose (>6.28) is considerably larger than collagen (<1.8, Fig. 2a). Finally, all bubbles nucleated in agarose, gelatin, and pure water were fully collapsed, e.g., *r*_0_ = 0, after completion of each impact.

So far, we have experimentally shown that (1) $({\lambda }_{max}-1)\propto {h}_{b}^{2}|{a}_{amp}|$ and under these conditions that (2) bubble bursts do not occur for relatively large bubbles (~0.5 mm) and (3) the critical acceleration increases with increasing concentration (or equivalently stiffness) of gels. The first conclusion, i.e., mechanical impacts result in pressure gradients in a soft gel, has a key application toward characterization of acceleration-induced pressure (*p*) in soft material samples. Currently, *p* ∝ *ah* (see Eq. S3 in the supplementary document) is often used to estimate *p* where *a* and *h* are acceleration and a coordinate of the sample in the impact direction because directly measuring *p* in pure water^24^ and soft gels^15^ during impact is still experimentally challenging. In this regard, we theoretically consider below how *p*, *λ* and *h* are linked to each other and show *p* ∝ *ah* is not a good estimation for the acceleration-induced pressure gradient for soft gels. It is worth noting that the Navier-Stokes derivations shown in the Supplementary Information are simplified, 1D, and just for water not a gel.

The other conclusions have important implications for biological studies investigating injury mechanisms due to rapid mechanical loading, e.g., possible traumatic brain injury mechanisms associated with impact or blast. As an example, air bubbles with a wide range of radii from several hundreds of micrometers to a few millimeters are often introduced into biosamples such as soft gels or brain tissues to mimic bubble-brain interplay *in vitro*^43^. However, because key characteristics of bubble dynamics in such soft materials are sensitive to the initial bubble size, use of relatively large bubbles may not realistically capture the bubble-brain interplay when a human head is exposed to impact or blast. In addition, because target organs, e.g., brain or skin, could have significantly different material properties, quantitative characterization of cavitation properties for different soft biosamples becomes directly relevant for developing reliable cavitation criteria in the scope of cavitation-induced injuries. Next we theoretically consider the important role of initial bubble size and gel stiffness on bubble dynamics in a soft gel and discuss possible mechanisms that capture the key experimental observations above.

### Theoretical Framework

**Figure 4:**
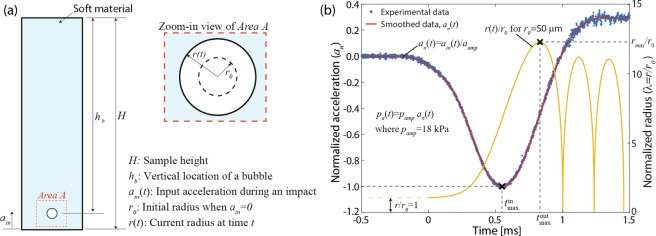
(**a**) Schematic of a spherical air bubble in a soft material sample. (**b**) Transient dynamics of a bubble for the initial radius *r*_0_ = 50 μm due to external acceleration (*a*_*in*_) induced by mechanical impact.

Consider a bubble in a soft material sample in Fig. 4a with an emphasis on the pressure and bubble size relation in a soft material sample. Upon mechanical impact, the sample is rapidly accelerated by *a*_*in*_ that results in acceleration-induced pressure (*p*_*a*_) in the sample. Based on the experimental results above, *p*_*a*_ is a function of time (*t*), the amplitude of input acceleration (*a*_*amp*_), and the bubble location (*h*_*b*_) in a gel. In the following analysis, we assume that bubble radius (*r*) is relatively small such there is no pressure gradient from the top of the bubble to its bottom, i.e., *p*_*a*_(*t*, *a*_*amp*_, *h*_*b*_) ≈ *p*_*a*_(*t*, *a*_*amp*_, *h*_*b*_ ± *r*). We also assume constant temperature and an incompressible material model, i.e., viscoelastic neo-Hookean material. These are reasonable assumptions for bubble expansion, our main focus, because a rate of change of bubble radius during bubble expansion is typically much slower than bubble collapse.

In fluid mechanics, the dynamics of a spherical bubble in Newtonian liquids has been well understood utilizing the Rayleigh–Plesset equation^44^. Unlike Newtonian liquids, soft gels have material stiffness and generally relax to an original configuration after loading, i.e., a Kelvin-Voigt viscoelastic model^13^. The Rayleigh–Plesset equation has been modified by incorporating the Kelvin-Voigt and neo-Hookean models^13,39^ to theoretically capture bubble dynamics in a viscoelastic medium as follows. The governing equation for the dynamics of a spherical bubble at *h*_*b*_ in Fig. 4a can be written as^13,44^

1 $$\frac{{p}_{v}-{p}_{a}(t,{a}_{amp},{h}_{b})}{\rho }+\frac{{p}_{G0}}{\rho }{(\frac{{r}_{0}}{r})}^{3k}=r\ddot{r}+\frac{3}{2}{\dot{r}}^{2}+\frac{4\nu \dot{r}}{r}+\frac{2\gamma }{\rho r}+\frac{\,\mu }{2\rho }[5-4\frac{{r}_{0}}{r}-{(\frac{{r}_{0}}{r})}^{4}]$$

where *p*_*G*0_ (=*p*_∞_(0) − *p*_*v*_(*T*_∞_) + 2*γ*/*r*_0_) is the initial partial pressure of the contaminant gas in a bubble, *p*_*v*_ is vapor pressure, *k* is the polytropic index (*k* = 1 for constant temperature), *T*_∞_ is the ambient temperature, *ρ*, *μ*, *ν* and *γ* are the density, shear modulus, viscosity, and surface tension of the sample, respectively, and *r*_0_ and *r*(*t*) are initial and current radius of the bubble, respectively, and the overdot denotes the derivative with respect to time. Recent studies that compare various Rayleigh-Plasset-like questions show that the Kelvin-Voigt and neo-Hookean models match well with experimental measurements on cavitation bubbles in soft gels^14^.

Equation 1 can be readily solved by using numerical solvers (see the Methods section) for the given initial conditions, $r(0)={r}_{0}\,{\rm{and}}\,\dot{r}(0)=0.$ For use of reasonable mechanical inputs that are directly relevant to actual impact, *p*_*a*_(*t*, *a*_*amp*_, *h*_*B*_) = *p*_*amp*_(*a*_*amp*_, *h*_*b*_)*a*_*n*_(*t*) is used as a driving force where *a*_*n*_(*t*) is normalized acceleration signal measured during an impact experiment and *p*_*amp*_ is the maximum amplitude of *p*_*a*_ at $t={t}_{max}^{in}$ (Fig. 4b, vertical axis on the left). The nominal material properties used in the numerical studies are summarized in Table S6 in Supplementary document.

One representative result in Fig. 4b (yellow line, vertical axis on the right) shows the transient dynamics of an air bubble (*r*_0_ = 50 μm) due to *p*_*a*_(*t*) (red line, vertical axis on the left) where *p*_*amp*_ = 18 kPa at $\,{t}_{max}^{in}=0.547\,{\rm{ms}}$. The bubble radius gradually increases in $0\le t\le {t}_{max}^{out}$where the “x” marker indicates the maximum radius *r*_*max*_ at $t={t}_{max}^{out}$. Then, although it is beyond our main focus, for $t > {t}_{max}^{out}$ the bubble rapidly collapses, i.e., *dr*/*dt* → − ∞ as *r*/*r*_0_ → 0, followed by bubble radius oscillation and, as a result, theoretical analysis of bubble collapse must appropriately account for change in temperature and compressibility of material.

**Figure 5:**
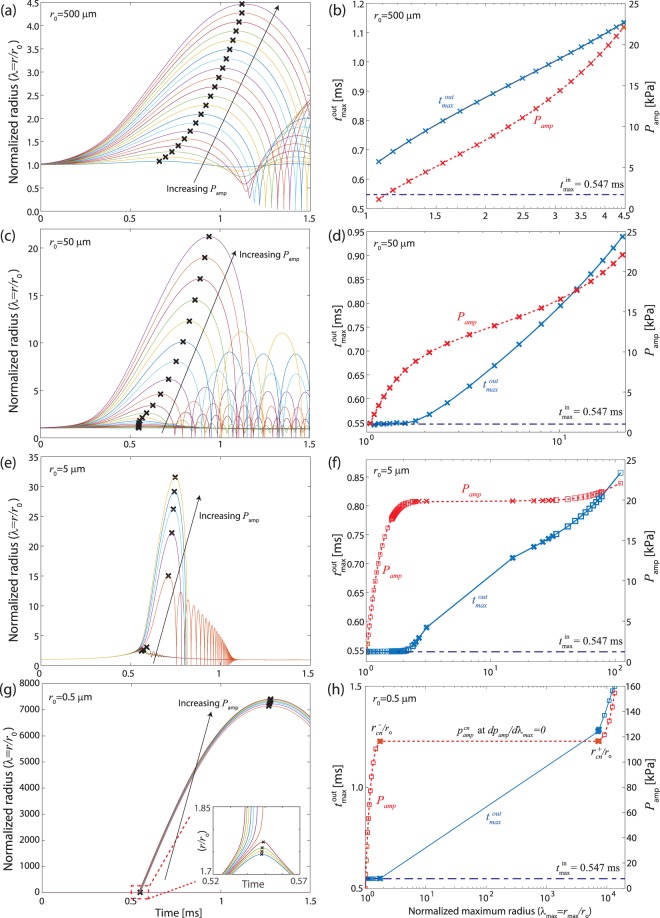
Numerical simulation to predict transient, dynamic response of an air bubble to impact. (**a**,**c**,**e** and **g**) The normalized bubble radius (*λ*(*t*) = *r*(*t*)/*r*_0_) for a different initial bubble radius, i.e., *r*_0_ = 500, 50, 5, and 0.5 μm, respectively, with linearly increasing amplitude of input pressure (*p*_*amp*_). “x” markers indicate the normalized maximum radius (*λ*_*max*_ = *r*_*max*_/*r*_0_) at $t={t}_{\max }^{out}$ for a given *p*_*amp*_. (**b**,**d**,**f**, and **h**) summarize how ${t}_{\max }^{out}$ and *r*_*max*_ (vertical axis on the left) as well as *p*_*amp*_ and *r*_*max*_ (vertical axis on the right) are correlated based on the results from a, c, e, and g, respectively.

To study the effect of initial bubble size on the bubble dynamics, we consider various *r*_0_ ranging from 0.5 to 500 μm. The left column of Fig. 5 shows the time evolution of the normalized bubble size (*λ*) with increasing input pressure (*p*_*amp*_). The initial bubble radii (*r*_0_) in a, c, e, and g are *r*_0_ = 500, 50, 5, and 0.5 μm, respectively. For each *r*_0_, the normalized bubble radius (*λ*(*t*) = *r*(*t*)/*r*_0_) is shown in time for a given *p*_*amp*_, which increases linearly. Note that “x” markers indicate the normalized maximum radius (*λ*_*max*_ = *r*_*max*_/*r*_0_) at $t={t}_{max}^{out}$ for a given *p*_*amp*_. The results in the left column are summarized in b, d, f, and h to show how ${t}_{max}^{out}$ and *λ*_*max*_ (vertical axis on the left) as well as *p*_*amp*_ and *λ*_*max*_ (vertical axis on the right) are correlated.

In general, *λ*_*max*_ and ${t}_{max}^{out}$ in the left column of Fig. 5 increase with increasing *p*_*amp*_; however, their specific trends strongly depend on *r*_0_. For relatively large initial radius (*r*_0_ = 500 μm, Fig. 5a,b), the effect of surface tension, which is inversely proportional to bubble size (see Eq. 1), becomes less important and, as a result, the maximum bubble radius (*r*_*max*_/*r*_0_) as well as the time (${t}_{max}^{out}$) corresponding to *r*_*max*_ smoothly increase as a function of *p*_*amp*_. As an example, the maximum radius vs *p*_*amp*_ plot for *r*_0_ ≥ 500 μm (Fig. S1 in the supplementary document) can be reasonably well captured by a linear approximation. Because the maximum bubble size for *r*_0_ = 0.52 mm is linearly proportional to amplitude of input acceleration as well as input pressure, we conclude that ${p}_{amp}\propto {h}_{b}^{2}|{a}_{amp}|$, in contrast to the commonly assumed *p* ∝ *ha* (see the supplementary document for details).

On the other hand, the slope of the ${t}_{max}^{out}$and *λ*_*max*_ curves is about zero within 1 ≤ *r*/*r*_0_ < 1.1 for *r*_0_ = 50,5, and 0.5 μm in Fig. 5d,f,h, respectively. This result indicates that input acceleration and bubble response are in phase, i.e., ${t}_{max}^{out}\approx {t}_{max}^{in}$, likely due to smaller inertial effects. Then a sharp increase in *λ*_*max*_ is observed in the *p*_*amp*_ − *λ*_*max*_ curves for *r*_0_ = 0.5 and 5 μm (Fig. 5f,h, respectively) near *λ*_*max*_ ≈ 1.1 even with a small increase in *p*_*amp*_, which is the key signature of the bubble burst^44,45^. For example, *λ*_*max*_ for *r*_0_ = 0.5 μm (see Fig. 5h) increases by 3 orders of magnitude from ${r}_{cn}^{-}/{r}_{0}$ to ${r}_{cn}^{+}/{r}_{0}$ at ${p}_{amp}^{cn}$ where ${p}_{amp}^{cn},$ the critical amplitude of pressure for cavitation nucleation, can be obtained by *dp*_*amp*_/*dλ*_*max*_ = 0 at $r={r}_{cn}^{-}$ and $={r}_{cn}^{+}$. This rapid expansion of a bubble is observed only for small *r*_0_ because of the transition in the dominant mechanism of material response from surface tension to inertia with increasing *r*.

The results of the numerical simulations match well with the experimentally observed, dynamic response of bubbles in a soft material. First, bubble bursts are indeed sensitive to initial bubble size. Experimentally we have observed bubble bursts in agarose, gelatin, and pure water likely with submicron-scale bubbles^40–42^, but no bubble bursts in collagen with macro bubbles (*r*_0_ ~ 0.5 mm), as shown above. Also it is confirmed that the use of macro bubbles for quantitative characterization of acceleration-induced pressure gradients in the sample offers a unique advantage because of a simple relation between pressure and bubble size without rapid bubble bursts.

On the contrary, the size of the small-scale bubbles becomes increasingly unstable when approaching the critical pressure (${p}_{amp}^{cn}$). As a result, experimental establishment of the *p*_*a*_ and *r* relation near ${p}_{amp}^{cn}$ becomes impractical. In addition, the simulation predicts that small bubble bursts are accompanied by a sharp increase in radius, which we also experimentally observed. As an example, assuming the initial bubble size is in the range of hundreds nanometer^40^ as discussed earlier, *λ*_*max*_ for the agarose sample in Fig. 3a becomes 10^3^~10^4^ due to the millimeter-scale *r*_*max*_, consistent with the numerical prediction in Fig. 5h.

It is worth noting that unlike rapid bubble expansion, the numerical studies predict that bubble collapse occurs regardless of the initial radius (*r*_0_) when the maximum bubble radius is sufficiently larger than *r*_0_ (see the left column of Fig. 5). This prediction matches with experimental observations (see Fig. S2 and Supplementary Movies 6–9). When impact occurs, bubbles expand during which significant energy is stored in the gel, i.e., surface energy and elastic energy associated with the material deformation. Upon the completion of the impact, input acceleration rapidly decreases and, as a result, the relaxation of the stored energy becomes dominant force that drives bubble collapse.

As discussed earlier, experimental measurements to quantify surface tension and elasticity of soft material samples, in particular, at fast loading conditions are still very limited. In this regard, although our numerical studies above using nominal soft material properties have revealed important characteristics of bubble dynamics in the sample, the current lack of reliable material property data makes quantitative predictions difficult. Therefore, in the following studies we perform non-dimensional analysis to further investigate bubble dynamics in the dimensionless parameter space of material properties.

By introducing $\lambda (\tilde{t})=r(\tilde{t})/{r}_{0},$ $\tilde{r}={r}_{0}/{r}_{cr,w}$, and $\tilde{t}=t/{t}_{max}^{in}$ Eq. 1 can be nondimensionalized as follows

2 $${\tilde{r}}^{3}{\mathscr{A}}(\lambda \ddot{\lambda }+\frac{3}{2}{\dot{\lambda }}^{2})+\tilde{r}[2 {\mathcal B} \frac{\dot{\lambda }}{\lambda }+\frac{1}{2}(5-\frac{4}{\lambda }-\frac{1}{{\lambda }^{4}})-{\tilde{p}}_{v}(1-\frac{1}{{\lambda }^{3}})+{\tilde{p}}_{a}]+{\mathscr{C}}(\frac{1}{\lambda }-\frac{1}{{\lambda }^{3}})=0$$

where ${\mathscr{A}}=\rho {({r}_{cr}/{t}_{max}^{in})}^{2}/\mu$, ${\mathcal B} =2(\nu \rho )/\mu {t}_{max}^{in}$, ${\mathscr{C}}=2\gamma /{r}_{cr}\mu$, ${\mathop{p}\limits^{ \sim }}_{v}(\mathop{t}\limits^{ \sim })={p}_{v}(\mathop{t}\limits^{ \sim })/\mu ,{\mathop{p}\limits^{ \sim }}_{a}(\mathop{t}\limits^{ \sim })={p}_{a}(\mathop{t}\limits^{ \sim })/\mu$, and *r*_*cr*, *w*_ is the critical radius of pure water at the onset of rapid bubble expansion or equivalently at bubble burst. Note that ${\mathscr{A}}, {\mathcal B} ,\,{\rm{and}}\,{\mathscr{C}}$, which are normalized by elastic modulus, are associated with inertia (Cauchy number), viscosity (Deborah number), and surface tension (Weber number) terms, respectively. For perturbation analysis of Eq. 2, we apply small input pressures $\tilde{p}$_*a*_($\tilde{t}$) = $\tilde{p}$*q*($\tilde{t}$) to the bubble where $\tilde{p}$ is the amplitude of the non-dimensionalized pressure and 0 < |*q*($\tilde{t}$)| ≪ 1. Upon the application of the input pressure, the bubble oscillates in time about the initial radius *r*_0_, which can be written as *λ*($\tilde{t}$) = *λ*_0_(1 + *f*($\tilde{t}$)) where 0 < |*f*($\tilde{t}$)| ≪ 1. By substituting the small input pressure and corresponding radius to Eq. 2, we obtain

3 $${\mathscr{A}}{\tilde{r}}^{3}\ddot{f}+2 {\mathcal B} \tilde{r}\dot{f}+(2{\mathscr{C}}+\tilde{r}(4-3{\tilde{p}}_{v}))f=-\,\tilde{p}q$$

The non-dimensional frequency of damped vibration (${\tilde{\omega }}_{d}$) for the bubble can be written as

4 $${\tilde{\omega }}_{d}=\frac{{\omega }_{d}}{{t}_{max}^{in}}=\frac{1}{\tilde{r}}{((4-\frac{3{p}_{v}}{\mu })\frac{\mu }{\rho {r}_{cr}^{2}}+\frac{4\gamma }{\rho {r}_{cr}^{3}}(\frac{1}{\tilde{r}})-\frac{4{\nu }^{2}}{{r}_{cr}^{4}}{(\frac{1}{\tilde{r}})}^{2})}^{\frac{1}{2}}\,$$

Note that *ω*_*d*_ increases with increasing elastic modulus and surface tension while decreasing with increasing viscosity. It is worth mentioning that when the size of pre-existing bubbles or defects at cavitation nucleation sites is much smaller than the critical size of pure water, i.e., $\tilde{r}$ ≫ 1, the last term with *ν* in Eq. 4 becomes insignificant due to the $\tilde{r}$^−2^ term and, as a result, *ω*_*d*_ ≈ *ω*_*N*_ where *ω*_*N*_ is the natural frequency of the bubble. This result indicates that the effect of viscosity is not critical for dynamics of submicron-scale bubbles. Furthermore when *ν* ≈ 0, Eq. 2 can be rewritten as^13,44^

5 $$\dot{\lambda }={(\frac{\mu }{\rho })}^{\frac{1}{2}}\frac{{t}_{max}^{in}}{\tilde{r}{r}_{cr,w}}{(\frac{2{\rm{\Delta }}p}{3\mu }(1-\frac{1}{{\lambda }^{3}})+\frac{2{p}_{Go}}{\mu {\lambda }^{3}}\mathrm{log}\lambda -\frac{2\gamma }{\mu {r}_{cr,w}}\frac{1}{\tilde{r}\lambda }(1-\frac{1}{{\lambda }^{2}})+\frac{1}{3}{{\mathbb{E}}}^{\ast })}^{\frac{1}{2}}\,$$

where Δ*p* = *p*_*v*_ − *p*_*a*_ and ${\mathbb{E}}$^*^ = −5 + 6*λ*^−1^ + 2*λ*^−3^ − 3*λ*^−4^. When a sufficiently large *p*_*a*_, i.e., ${p}_{amp} > {p}_{amp}^{cr}$, is applied to the small bubble or defect, we have *λ*(*t*) = *λ*_*max*_ ≫ 1 due to bubble bursts and $\dot{\lambda }=0$ at $t={t}_{max}^{out}$. Using these known conditions at $t={t}_{max}^{out}$, the first order approximation of Eq. 5 becomes

6 $${\lambda }_{max}=\frac{3}{2}(\frac{2\gamma }{{r}_{0}\mu }-2)/(\frac{{\rm{\Delta }}p}{\mu }-\frac{5}{2})\,$$

**Figure 6:**
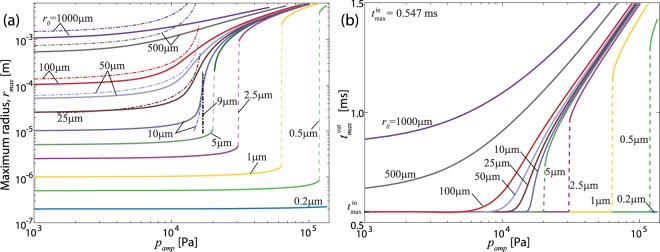
(**a**) The maximum radius (*r*_*max*_) and (**b**) corresponding time ($${t}_{max}^{out})$$ as a function of the amplitude of acceleration-induced input pressure (*p*_*amp*_) for an air bubble with an initial radius ranging from 0.2 μm to 1000 μm. Dash-dot lines and solid/dashed lines are from the first order approximation (Eq. 6) and directly numerical simulation (Eq. 1), respectively.

In Fig. 6a, the result of Eq. 6 (dash-dot lines) for the nominal material sample in Table S6 is directly compared with the numerical simulation (solid and dashed lines) for 20 ≥ *λ* ≥ 1 and 20 kPa ≥ *p*_*amp*_ ≥ 1 kPa. For relatively large *r*_0_ (≥9 μm), the first order approximation matches with the direct simulation reasonable well. In addition, Equation 6 correctly predicts the critical *r*_0_ and *p*_*amp*_ that bound the two distinctive regimes with and without bubbles burst. First, from the denominator of Eq. 6, *λ*_*max*_ → ∞, the key signature of bubble burst, as Δ*p*/*μ* → 5/2, i.e., Δ*p*_*cr*_ = 20 kPa. Second, *λ*_*max*_ < 0 in Eq. 6 when *r*_0_ < *γ*/*μ* and, as a result, the sum of all terms within the square root in Eq. 5 becomes negative. Therefore, ${r}_{0}^{cr}={r}_{0}={\gamma }/{\mu }$ is a bifurcation point from real to imaginary number solutions for $\dot{\lambda }$, i.e., ${r}_{0}^{cr}=9\,{\rm{\mu }}{\rm{m}}.$

Note that Eq. 6 fails to capture the *p*_*amp*_ and *λ*_*max*_ relation for ${r}_{0}^{cr} < 9\,{\rm{\mu }}{\rm{m}}$ because rapid changes in bubble size due to bubble bursts cannot be appropriately captured by the first order approximation. In other words, the effect of the viscosity on bubble expansion must be appropriately considered after bubble burst. The same conclusion can be made from Eq. 4 because ${\tilde{\omega }}_{d}$ is very sensitive to bubble size for relatively large bubbles due to the $\tilde{r}$^−2^ term. Equation 3 could be utilized, e.g., Fig. 6, for analytical prediction of the critical acceleration. However, Eq. 3 still requires a reasonable *r*_0_ estimate while direct measurement of the submicron-scale bubbles or material flaw remains very challenging experimentally.

While we have used a deterministic model to study cavitation behavior in soft gel, experimental measurements on the critical acceleration for cavitation nucleation are distributed over a broad range for a given stiffness of soft gel samples. This suggests that cavitation nucleation is stochastic in nature and, as a result, developing a theoretical frame work that capture stochastic distribution of the critical mechanical input at the onset of cavitation nucleation would be interesting future work and we believe can be guided by the distributions of data obtained using our technique.

## Conclusion and Discussion

Soft gels that mimic properties of biological samples are increasingly utilized as tissue simulants for the assessment of potential damage, e.g., blunt injuries, to a human body against rapid mechanical inputs. As a result, capabilities to accurately characterize and predict transient, dynamic response of soft materials under fast loading rate conditions have become critical for such biomedical applications. In this regard, we have performed experimental and theoretical investigations on (1) acceleration-induced pressure gradients in biologically-relevant soft gels during impact and (2) the corresponding critical transition in the material response from small deformation to a sudden bubble burst.

We have characterized collagen samples with macro air bubbles (about 0.5 mm in radius) and monitored the dynamic response of individual bubbles during impact. This study reveals that the maximum bubble radius (*r*_*max*_) monotonically increases as a function of amplitude of input acceleration (*a*_*in*_) and is also proportional to the square of the depth of individual bubbles (*h*_*b*_) in the sample, i.e., *r*_*max*_ ∝ *ah*^2^. This simple size-pressure relation offers an attractive advantage in utilizing large bubbles for experimental, quantitative characterization of acceleration-induced pressure gradients. We have also considered agarose samples without the macro air bubbles and quantified the critical acceleration that triggers the onset of bubble bursts, which increases with increasing gel stiffness. Finally, we have found that bubble bursts are sensitive to the initial bubble size by comparing experimental data for different types of gels with and without macro bubbles.

To explain these key experimental observations, we have developed theoretical framework. The model predicts the onset of bubble bursts can be triggered only when the initial bubble size is smaller than the critical size (about 10 micron). For micro/nanoscale bubbles (<10 μm), their size becomes unstable as it approaches a critical radius due to the transition from surface tension to inertia dominant material deformation. The model also shows that the maximum radius and amplitude of input pressure (*p*_*amp*_) are linearly correlated for larger bubbles (>500 μm). Based on ${r}_{max}\propto {a}_{in}{h}_{b}^{2}$ and ∝*p*_*amp*_ relations, we empirically determine that the pressure gradient in a soft material sample during impact is simply ${p}_{amp}\propto {a}_{in}{h}_{b}^{2}$.

Our study on how biological soft materials respond to rapid mechanical inputs is an important step to understanding possible underlying injury mechanism(s) in blunt trauma and cavitation-induced brain injury. As an example, cavitation is being conjectured as a possible mechanism for mild to moderate traumatic brain injury (mTBI)^46–48^. However, the current understanding of dynamic cavitation in biological materials, e.g., brain tissues, is still very limited and, as a result, a range of mechanical impact that is relevant to cavitation-induced brain injury is still unknown. To address the current limitation, we have used collagen, agarose, and gelatin as a tissue simulant and have experimentally considered the acceleration-induced pressure gradient in the simulant and quantified the critical acceleration corresponding to cavitation nucleation. This quantitative measurements on the cavitation properties can be utilized for developing injury criteria for cavitation-induced TBI.

In addition, our experimental approaches have the potential for clinically- and biologically-relevant studies that require use of 3-dimentional (3D) extracellular matrix (ECM) such as collagen and agarose. As an example, a reasonable 3D *in vitro* model that biologically and mechanically represents target organs could be developed by culturing fibroblast or neurons in collagen/agarose at a specific concentration to match known mechanical stiffnesses of skin or brain, respectively. Biological studies that utilize well-designed 3D cell-ECM systems and mechanical inputs directly relevant to blunt injuries would provide unique opportunities to probe the specific cell-ECM interplay during impact and shed light on key mechanism(s) in various forms of traumatic injuries.

## Methods

### Materials

For 1% [g/ml] collagen samples (Collagen Type I, Rat Tail, Corning, Bedford, MA), we prepare 24 ml of ice cold collagen solution by mixing 2.4 ml of Sterile 10X phosphate buffered saline, 8.1 μl of 1 N NaOH, and 18 ml of sterile deionized water with 7 ml of collagen solution at 3.42 mg/ml. Then the solution is thoroughly mixed using a 10-ml pipette and vortex mixer to have randomly distributed bubbles in the sample. Immediately after the mixing, 4 ml of 1% collagen solution is added to six individual transparent cuvettes using a 10 ml pipette tip and cured in an incubator overnight.

For 0.3%, 0.58%, 0.9%, and 1.5% [g/ml] agarose samples, we add 80 ml of room temperature water into a Pyrex beaker with a magnetic stir bar. Then put the beaker on a magnetic stirrer and slowly add the required amount of agarose powder (0.24, 0.46, 0.72, and 1.2 g for 0.3%, 0.58%, 0.9%, and 1.5% agarose, respectively) into the beaker to avoid the formation of clusters. Measure the total weight of the beaker and solution before heating and boil the solution for 10 mins with a Pyrex beaker cover while stirring. To compensate evaporation, measure the weight of the beaker and add water to bring it back to its initial weight. After cooling the beaker for about 10–20 minutes, insert 4 ml of agarose solution into the individual eighteen cuvettes using a 10 ml pipette and cure them at room temperature overnight.

### Experimental setup

The integrated drop tower system consists of a conventional drop tower impact system (Dynatup 9210, Instron, Norwood, MA), two high speed cameras (Fastcam SA-X2RV, Photron, San Diego, CA), a cuvette and holder, accelerometers, and a data acquisition system. Soft gel samples are prepared inside standard plastic cuvettes for ease of handling. Each cuvette is sealed with a cap and then glued onto the cuvette holder which consists of two horizontal plates connected by four vertical columns.

For optical observation of samples, the two independent high speed cameras are mounted on two camera stands such that the cameras can concurrently capture the front and side views of the cuvette, typically at 25 k or 50 k frames per second (fps). Data acquisition is performed using a system consisting of an NI PXIe-8135 embedded controller and an NI PXI-6115 multifunction I/O module using SignalExpress 2014 data acquisition software (all from National Instruments Corp., Austin, TX). A three-axis ICP-based accelerometer (model #356A01, PCB Piezotronics, Depew, NY) is connected to three channels of the data acquisition system through an ICP signal conditioner (model #480B21, PCB Piezotronics). Data are acquired at a rate of 1 MHz and triggered off the vertical axis of the accelerometer. More details on the experimental setup and procedure can be found elsewhere^10,15^.

### Image analysis for bubble dynamics

For experimental measurement of bubble size during impact, high-speed images are analyzed at each frame (25,000 or 50,000 frames/sec) using *ImageJ*. To differentiate bubbles from the background during the image analysis, the same threshold setting is used for all image frames. All pixels that are above the threshold due to bubbles are counted and converted to the corresponding area. Then, radius of each bubble is calculated using *r* = (*A*/*π*)^1/2^ where *A *is the projected area of the bubbles. Following the same procedure, we also monitor the effective radius of a solid structure, *reference*, at the bottom of a cuvette.

### Statistical analysis for experimental data

For the statistical analysis, *mean* and *std* functions of Matlab are utilized to compute the mean and standard deviation of experimental data.

### Numerical simulation and optimization

*ODE45* function of Matlab are used to simulate dynamic response of bubbles in a soft gel (Eq. 1).

## Electronic supplementary material

Supplement document

Movie1

Movie2

Movie3

Movie4

Movie5

Movie6

Movie7

Movie8

Movie9

## References

[1] Jussila J, Leppaniemi A, Paronen M, Kulomaki E (2005). Ballistic skin simulant. Forensic Sci Int.

[2] Wen YK, Xu C, Jin YX, Batra RC (2017). Rifle bullet penetration into ballistic gelatin. J Mech Behav Biomed.

[3] Falland-Cheung L (2017). Use of agar/glycerol and agar/glycerol/water as a translucent brain simulant for ballistic testing. J Mech Behav Biomed.

[4] Crisco JJ (2012). Magnitude of Head Impact Exposures in Individual Collegiate Football Players. J Appl Biomech.

[5] Morse JD, Franck JA, Wilcox BJ, Crisco JJ, Franck C (2014). An Experimental and Numerical Investigation of Head Dynamics Due to Stick Impacts in Girls' Lacrosse. Ann Biomed Eng.

[6] Meaney David F., Morrison Barclay, Dale Bass Cameron (2014). The Mechanics of Traumatic Brain Injury: A Review of What We Know and What We Need to Know for Reducing Its Societal Burden. Journal of Biomechanical Engineering.

[7] Dimitriadis EK, Horkay F, Maresca J, Kachar B, Chadwick RS (2002). Determination of elastic moduli of thin layers of soft material using the atomic force microscope. Biophys J.

[8] Raub CB, Putnam AJ, Tromberg BJ, George SC (2010). Predicting bulk mechanical properties of cellularized collagen gels using multiphoton microscopy. Acta Biomater.

[9] Moreno-Arotzena O, Meier JG, del Amo C, Garcia-Aznar JM (2015). Characterization of Fibrin and Collagen Gels for Engineering Wound Healing Models. Materials.

[10] Kang Wonmo, Chen YungChia, Bagchi Amit, O’Shaughnessy Thomas J. (2017). Characterization and detection of acceleration-induced cavitation in soft materials using a drop-tower-based integrated system. Review of Scientific Instruments.

[11] Zimberlin JA, Sanabria-DeLong N, Tew GN, Crosby AJ (2007). Cavitation rheology for soft materials. Soft Matter.

[12] Kundu S, Crosby AJ (2009). Cavitation and fracture behavior of polyacrylamide hydrogels. Soft Matter.

[13] Gaudron R., Warnez M. T., Johnsen E. (2015). Bubble dynamics in a viscoelastic medium with nonlinear elasticity. Journal of Fluid Mechanics.

[14] Estrada JB, Barajas C, Henann DL, Johnsen E, Franck C (2018). High strain-rate soft material characterization via inertial cavitation. J Mech Phys Solids.

[15] Kang, W., Adnan, A., O’Shaughnessy, T. & Bagchi, A. Cavitation nucleation in gelatin: Experiment and mechanism. *Acta Biomater* (2017). 10.1016/j.actbio.2017.11.030 29191509

[16] Normand V, Lootens DL, Amici E, Plucknett KP, Aymard P (2000). New insight into agarose gel mechanical properties. Biomacromolecules.

[17] Kwon J, Subhash G (2010). Compressive strain rate sensitivity of ballistic gelatin. J Biomech.

[18] Webster, J. G. & Eren, H. Measurement, Instrumentation, and Sensors Handbook: Spatial, Mechanical, Thermal, and Radiation Measurement, (CRC Press, 2014).

[19] Style, R. W., Hyland, C., Boltyanskiy, R., Wettlaufer, J. S. & Dufresne, E. R. Surface tension and contact with soft elastic solids. *Nat Commun* **4** (2013). 10.1038/ncomms3728 24201430

[20] Xu XJ, Jagota A, Paretkar D, Hui CY (2016). Surface tension measurement from the indentation of clamped thin films. Soft Matter.

[21] Xu, Q. et al. Direct measurement of strain-dependent solid surface stress. *Nat Commun* **8** (2017). 10.1038/s41467-017-00636-y PMC5601460 28916752

[22] Lopez-Garcia MDC, Beebe DJ, Crone WC (2010). Young's modulus of collagen at slow displacement rates. Bio-Med Mater Eng.

[23] McKee CT, Last JA, Russell P, Murphy CJ (2011). Indentation Versus Tensile Measurements of Young's Modulus for Soft Biological Tissues. Tissue Eng Part B-Re.

[24] Pan Z (2017). Cavitation onset caused by acceleration. Proc Natl Acad Sci.

[25] Grinnell F, Petroll WM (2010). Cell Motility and Mechanics in Three-Dimensional Collagen Matrices. Annu Rev Cell Dev Bi.

[26] Cukierman E, Pankov R, Yamada KM (2002). Cell interactions with three-dimensional matrices. Curr Opin Cell Biol.

[27] Bar-Kochba, E., Scimone, M. T., Estrada, J. B. & Franck, C. Strain and rate-dependent neuronal injury in a 3D in vitro compression model of traumatic brain injury. *Sci Rep-Uk* **6** (2016). 10.1038/srep30550 PMC4969749 27480807

[28] Markidou Anna, Shih Wan Y., Shih Wei-Heng (2005). Soft-materials elastic and shear moduli measurement using piezoelectric cantilevers. Review of Scientific Instruments.

[29] Mercey E (2010). The application of 3D micropatterning of agarose substrate for cell culture and in situ comet assays. Biomaterials.

[30] Balgude AP, Yu X, Szymanski A, Bellamkonda RV (2001). Agarose gel stiffness determines rate of DRG neurite extension in 3D cultures. Biomaterials.

[31] Lee SJ (2011). Optically based-indentation technique for acute rat brain tissue slices and thin biomaterials. J Biomed Mater Res B.

[32] Budday S (2015). Mechanical properties of gray and white matter brain tissue by indentation. J Mech Behav Biomed.

[33] Soza G (2005). Determination of the elasticity parameters of brain tissue with combined simulation and registration. Int J Med Robot.

[34] Gozna ER, Marble AE, Shaw AJ, Winter DA (1973). Mechanical properties of the ascending thoracic aorta of man. Cardiovasc Res.

[35] Gamonpilas C, Charalambides MN, Williams JG (2009). Determination of large deformation and fracture behaviour of starch gels from conventional and wire cutting experiments. J Mater Sci.

[36] Forte AE, D'Amico F, Charalambides MN, Dini D, Williams JG (2015). Modelling and experimental characterisation of the rate dependent fracture properties of gelatine gels. Food Hydrocolloids.

[37] Salisbury CP, Cronin DS (2009). Mechanical Properties of Ballistic Gelatin at High Deformation Rates. Exp Mech.

[38] Richler D, Rittel D (2014). On the Testing of the Dynamic Mechanical Properties of Soft Gelatins. Exp Mech.

[39] Movahed P, Kreider W, Maxwell AD, Hutchens SB, Freund JB (2016). Cavitation-induced damage of soft materials by focused ultrasound bursts: A fracture-based bubble dynamics model. J Acoust Soc Am.

[40] Ushikubo FY (2010). Evidence of the existence and the stability of nano-bubbles in water. Colloid Surface A.

[41] Tyrrell, J. W. G. & Attard, P. Images of nanobubbles on hydrophobic surfaces and their interactions. *Phys Rev Lett* **87** (2001). 10.1103/PhysRevLett.87.176104 11690285

[42] Fang, C. K., Ko, H. C., Yang, C. W., Lu, Y. H. & Hwang, I. S. Nucleation processes of nanobubbles at a solid/water interface. *Sci Rep-Uk* **6** (2016). 10.1038/srep24651 PMC4835695 27090291

[43] Hong Y, Sarntinoranont M, Subhash G, Canchi S, King MA (2016). Localized Tissue Surrogate Deformation due to Controlled Single Bubble Cavitation. Exp Mech.

[44] Brennen, C. E. Cavitation and bubble dynamics. (Cambridge University Press, 2014).

[45] Hutchens SB, Fakhouri S, Crosby AJ (2016). Elastic cavitation and fracture via injection. Soft Matter.

[46] Estrada JB (2017). Microcavitation as a Neuronal Damage Mechanism in an In Vitro Model of Blast Traumatic Brain Injury. Biophys J.

[47] Wu, Y. T. & Adnan, A. Effect of Shock-Induced Cavitation Bubble Collapse on the damage in the Simulated Perineuronal Net of the Brain. *Sci Rep-Uk* **7** (2017). 10.1038/s41598-017-05790-3 PMC5509702 28706307

[48] Kanagaraj J, Chen B, Xiao S, Cho M (2018). Reparative Effects of Poloxamer P188 in Astrocytes Exposed to Controlled Microcavitation. Ann Biomed Eng.

