# Supplementary material for: Acceleration-induced pressure gradients and cavitation in soft biomaterials
Source: Sci Rep. 2018 Oct 26;8:15840. doi: 10.1038/s41598-018-34085-4 (PMC6203720; doi:10.1038/s41598-018-34085-4)
Supplement: Supplementary file 1 — Supplement document [file 41598_2018_34085_MOESM1_ESM.pdf]

Acceleration-induced pressure gradients and cavitation in soft biomaterials

Wonmo Kang[1,*] and Marc Raphael[2]
[1]Leidos, Inc., Arlington, VA 22203, USA
[2]Naval Research Laboratory, Washington, DC 20375, USA
*Email: wonmo.kang.ctr.ks@nrl.navy.mil

Supplement

1. Bubble size and location

Table S1 Summary of bubble size and location.

Bubble index	1	2	3	4	5	6	7	8	Ref
Initial radius (r_o) [mm]	0.52	0.56	0.51	0.50	0.49	0.50	0.53	0.52	0.66
Vertical location (h_b) [mm]	10.7	10.9	14.8	15.8	17.1	22.9	28.1	29.2	36.0

2. Numerical simulation

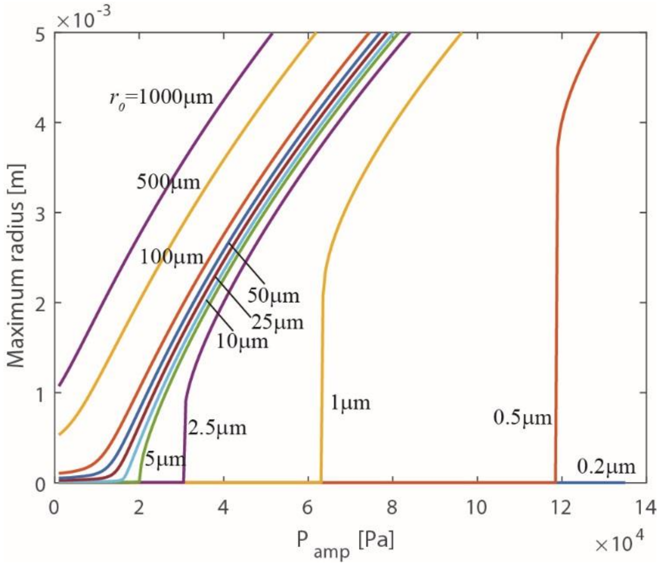

Figure S1: Theoretical prediction for the maximum radius for given p_{amp}. While a sudden increase in the maximum radius is predicted for smaller bubbles, a much simpler radius-pressure relation, which can be approximated by a linear relation, is observed for larger bubbles (≥0.5mm).

3. Bubble collapse for macro bubbles in collagen

For analysis of bubble collapse, we consider circularity, $C = 4\pi(\text{Area})/(\text{Perimeter})^2$, of the bubbles and reference (Figure S1a) because non-symmetric bubble collapse, which corresponds to smaller C values, is commonly observed in pure water [S1]. Within $t < 1.0$ ms, bubbles consistently have larger C values ($C_b > 0.9$) due to their spherical shape compared to the non-circular reference ($C_r \approx 0.5$). As expected, C_b significantly decreases for $h_{drop} > 8$ cm when $t > 1.0$ ms due to the non-symmetric bubble collapse (see Movies 1-5).

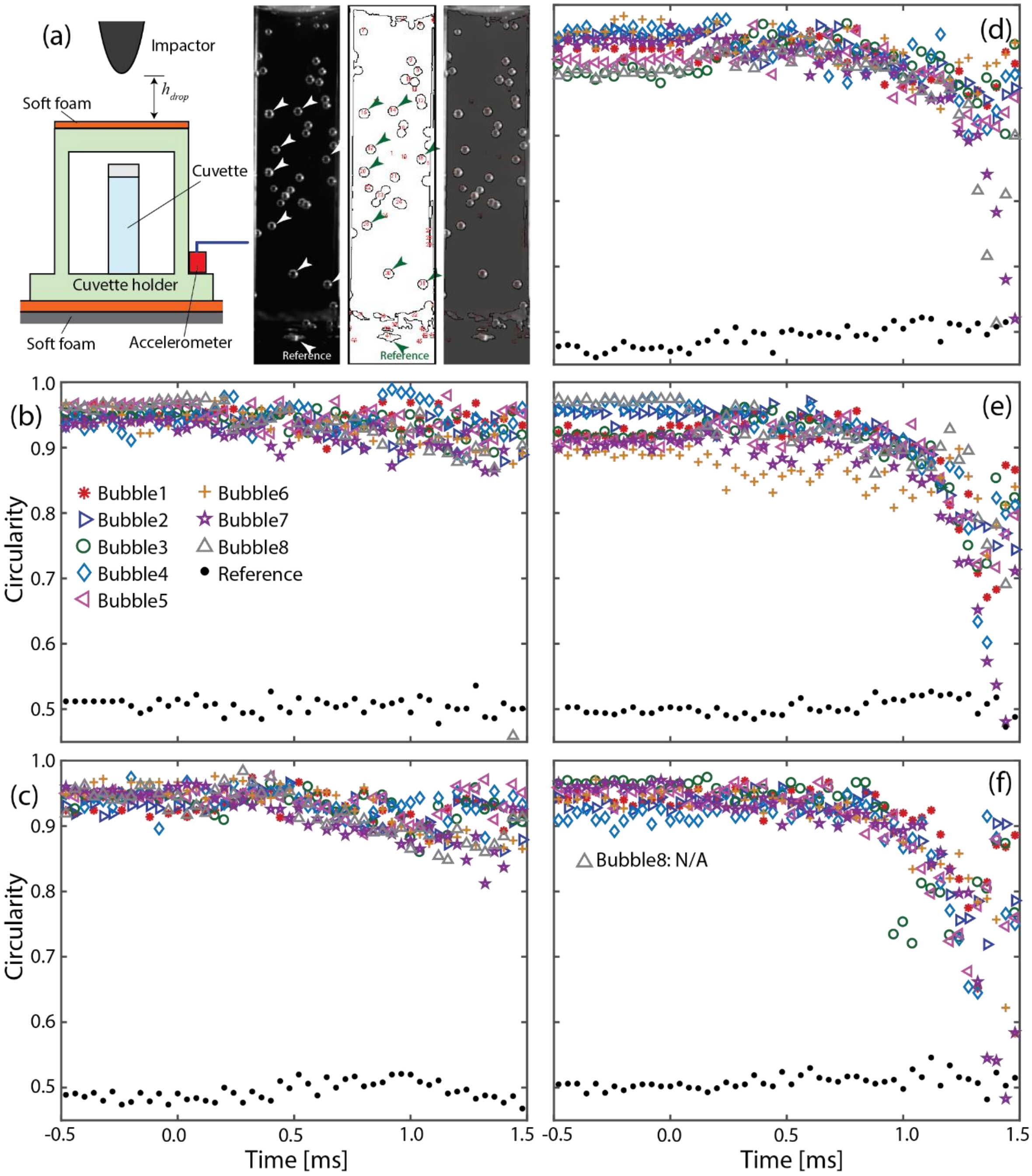

Figure S2 Circularity of air bubbles in a cuvette during a mechanical impact. (From left to right in a) Schematic of a sample holder under a drop-tower system for characterization of a soft material sample in a cuvette; a high speed camera image of air bubbles in a collagen sample, a processed image for bubble size and shape analysis during impact, and a merged image, respectively. The merged image of the high speed and masked images shows that the image analysis capture the actual size and shape of bubbles in the cuvette well. In (b)-(f), the circularity ($= 4\pi(Area)/(Perimeter)^2$) for *Bubble1-8* in a is quantified using an image processing program, *ImageJ*.

4. Experimental data for the critical acceleration of agarose samples

Table S2 Experimental data for 0.3% agarose sample

	Critical acceleration (a_{cr}) [g]	Sample number	Depth of cavitation bubble [m]	$a_{cr} \times h_B$ [g·m]
Cavitation at the first drop	554.69	2	0.033	18.54
	649.41	3	0.038	24.68
	540.04	4	0.038	20.52
	563.48	6	0.038	21.41
	577.15	8	0.038	21.93
	478.52	11	0.038	18.18
	462.89	13	0.024	11.23
	495.12	15	0.036	17.92
	604.49	17	0.038	22.97
Ave	547.31		0.036	19.71
Cavitation after multiple drops	722.66	1	0.036	25.74
	N/A	5	N/A	N/A
	744.14	7	0.028	20.95
	662.11	9	0.038	25.16
	657.23	10	0.033	21.53
	571.29	12	0.030	17.07
	560.55	14	0.036	19.93
	537.11	16	0.038	20.41
	N/A	18	N/A	N/A
Ave	636.44		0.034	21.54

Table S3 Experimental data for 0.58% agarose sample

	Critical acceleration (a_{cr}) [g]	Sample number	Depth of cavitation bubble [m]	$a_{cr} \times h_{B,i}$ [g·m]
Cavitation at the first drop	673.83	1	0.038	25.61
	717.77	2	0.036	25.50
	719.73	5	0.031	22.18
	629.88	6	0.029	18.40
	488.28	8	0.030	14.49
	687.50	9	0.029	19.68
	688.48	11	0.036	24.88
	690.43	13	0.022	15.37
	580.08	15	0.033	19.14
Ave	652.89		0.031	20.58
Cavitation after multiple drops	742.19	3	0.036	26.56
	836.91	4	0.027	22.91
	617.19	7	0.030	18.33
	733.40	10	0.028	20.81
	828.13	12	0.028	23.50
	862.30	14	0.035	30.06
	785.16	16	0.034	26.88
	757.81	17	0.036	27.56
	N/A	18	N/A	N/A
Ave	770.39		0.032	24.58

Table S4 Experimental data for 0.9% agarose sample

	Critical acceleration (a_{cr}) [g]	Sample number	Depth of cavitation bubble [m]	$a_{cr} \times h_{B,i}$ [g·m]
Cavitation at the first drop	817.38	1	0.033	26.58
	744.14	2	0.038	28.28
	641.60	8	0.038	24.38
	807.62	11	0.029	23.72
	600.59	18	0.038	22.82
	707.03	19	0.034	24.18
Ave	719.73		0.035	24.99
Cavitation after multiple drops	674.80	3	0.032	21.67
	555.66	4	0.038	21.12
	772.46	5	0.038	29.35
	600.59	6	0.027	16.22
	704.10	7	0.026	18.54
	822.27	9	0.028	23.17
	750.98	10	0.035	26.21
	667.97	12	0.034	23.02
	666.02	13	0.031	20.61
	718.75	14	0.038	27.31
	802.73	15	0.034	27.65
	798.83	16	0.038	30.36
	961.91	17	0.038	36.55
Ave	730.54		0.034	24.75

Table S5 Experimental data for 1.5% agarose sample

	Critical acceleration (a_{cr}) [g]	Sample number	Depth of cavitation bubble [m]	$a_{cr} \times h_{B,i}$ [g·m]
Cavitation at the first drop	817.38	3	0.038	31.06
	792.97	4	0.038	30.13
	815.43	8	0.038	30.99
	990.23	13	0.038	37.63
	982.42	14	0.038	37.33
	1097.66	18	0.038	41.71
Ave	916.02		0.038	34.81
Cavitation after multiple drops	839.84	1	0.038	31.91
	N/A	2	N/A	N/A
	1041.02	5	0.030	31.65
	832.03	6	0.033	27.85
	N/A	7	N/A	N/A
	N/A	9	N/A	N/A
	972.66	10	0.030	29.56
	1092.77	11	0.029	31.30
	N/A	12	N/A	N/A
	N/A	15	N/A	N/A
	N/A	16	N/A	N/A
	1155.27	17	0.038	43.90
Ave	988.93		0.033	32.69

5. Pressure gradient

For a pressure-acceleration relation, the incompressive Navier-Stokes equation can be written as

$$\rho \frac{DU}{Dt} = -\nabla P + \rho g + \eta \nabla^2 U \qquad \text{(Eq. S1)}$$

where U is the flow velocity, P is pressure, g is gravity, and η is viscosity. Assume that vertical velocity (u_x) is the only non-zero component in U and $u_x \approx 0$. Then (S1) can be simplified as $-dp_x/dx = \rho(a_x - g)$ where a_x is acceleration in x-direction and, as a result, the pressure drop can be written as

$$p_x(x) = \rho(a_x - g)h \qquad \text{(Eq. S2)}$$

where h is the vertical height of the sample. Finally, for $a_x \gg g$, Eq. (S2) can be written as

$$p_x(x) = \rho a_x h \qquad \text{(Eq. S3)}$$

6. Nominal material properties

Table S6 Nominal material properties used in the numerical analysis for dynamic response of bubbles

p_v [Pa]	ν [m^2/s]	ρ [kg/m^3]	γ [N/m]	μ [Pa][S2]
3170	8.817×10^{-7}	1000	72×10^{-3}	8000

[S1] Brennen CE. Cavitation and bubble dynamics. New York: Cambridge University Press; 2014.
[S2] Lopez-Garcia MDC, Beebe DJ, Crone WC. Young's modulus of collagen at slow displacement rates. Bio-Med Mater Eng 2010;20:361-9.
